# Evaluating the effects of the educational course on undergraduate students’ perceived knowledge, attitude, and motivation: a quasi-experimental study in Iran

**DOI:** 10.1186/s12909-026-09915-z

**Published:** 2026-07-22

**Authors:** Mehri Maleki, Bahram Amirshakeri, Ali Jahan, Hamid Soltani-zangbar

**Affiliations:** 1 Department of Audiology, Faculty of Rehabilitation Sciences, Tabriz University of Medical Sciences, Tabriz, Iran; 2 Department of Physiotherapy, Faculty of Rehabilitation Sciences, Tabriz University of Medical Sciences, Tabriz, Iran; 3 Department of Speech Therapy, Faculty of Rehabilitation Sciences, Tabriz University of Medical Sciences, Tabriz, Iran; 4 Department of Neuroscience and Cognition, Faculty of Advanced Medical Sciences, Tabriz University of Medical Sciences, Tabriz, Iran

**Keywords:** Rehabilitation education, Knowledge, Attitude, Motivation, Undergraduate students, Quasi-experimental design, Iran

## Abstract

**Background:**

The increasing global burden of chronic diseases, disability, and population aging highlights the growing need for well-prepared rehabilitation professionals. Educational experiences play an important role in shaping students’ perceived knowledge, attitudes, and motivation, which are key components of professional development. In Iran, many students enter rehabilitation programs with limited prior exposure to the field. This study aimed to evaluate the effect of the Principles of Rehabilitation course on undergraduate students’ perceived knowledge, attitude, and motivation, and to explore differences associated with early clinical exposure.

**Methods:**

A quasi-experimental pretest–posttest design was conducted among 184 undergraduate students in physiotherapy, occupational therapy, speech therapy, and audiology at Tabriz University of Medical Sciences. First-year students (*n* = 107) completed the Rehabilitation Sciences Knowledge, Attitude, and Motivation (RS-KAM) questionnaire before and after the course. Physiotherapy students who had not yet taken the course served as the control group. Third-year students (*n* = 77) completed a single assessment for cross-sectional comparison. The primary analysis compared change scores between intervention and control groups. Secondary analyses included within-group comparisons, cross-sectional analyses, and correlation assessments.

**Results:**

Between-group analysis showed a significant difference in attitude change, with the intervention group demonstrating a more positive shift compared with the control group (*p* = 0.001). No significant between-group differences were observed for perceived knowledge or motivation. In both groups, perceived knowledge increased over time, while motivation decreased. Cross-sectional comparisons indicated that third-year students reported higher perceived knowledge but lower motivation than first-year students. Positive correlations among perceived knowledge, attitude, and motivation were observed, particularly among first-year students.

**Conclusions:**

The *Principles of Rehabilitation* course was associated with improved student attitudes but did not result in greater gains in perceived knowledge or motivation compared with controls. These findings suggest that structured educational experiences may contribute to the development of professional attitudes, while perceived knowledge may be influenced by both formal and informal learning experiences. The observed decline in motivation highlights the importance of educational strategies that support students’ motivation alongside their academic and professional development.

**Supplementary Information:**

The online version contains supplementary material available at https://doi.org/10.1186/s12909-026-09915-z.

## Introduction

The global population aged 60 years and older is expected to double by 2050 [1]. http://www.un.org/en/sections/ issues-depth/ageing/index.html. Accessed December 24). Concurrently, the prevalence of non-communicable diseases continues to rise, often accompanied by long-term functional impairments and disabilities [2–5]. Compounding these challenges are injury-related conditions from traffic accidents, industrial incidents, and natural disasters, which further contribute to the global burden of chronic disability [6]. These demographic and epidemiological transitions emphasize the need for accessible, comprehensive, and well-resourced rehabilitation services that aim to restore function, foster independence, and promote social participation while reducing environmental barriers (https://www.who.int/news-room/fact-sheets/detail/rehabilitation, [7, 8]. Rehabilitation has been recognized as a crucial determinant of health system efficacy, with evidence demonstrating its potential to reduce hospital readmissions, lower healthcare costs, and improve workforce productivity [9], https://www.who.int/news-room/fact-sheets/detail/rehabilitation, [10]. Accordingly, the World Health Organization (WHO) has identified equitable access to rehabilitation as an essential component of universal health coverage and advocates for its integration across all tiers of healthcare delivery, including primary care (https://www.who.int/news-room/fact-sheets/detail/rehabilitation, [7]. In Iran, rehabilitation sciences—including physiotherapy, occupational therapy, speech therapy, audiology, optometry, orthotics and prosthetics, and rehabilitation management—are taught from undergraduate to doctoral levels under curricula approved by the Ministry of Health and Medical Education. Developing competent rehabilitation professionals remains a high priority, given the growing demand for skilled practitioners. Achieving this goal requires evidence-based educational strategies that cultivate theoretical knowledge, practical skills, professional identity, and motivation [11]. Constructivist learning theory asserts that learners actively construct knowledge through experience and reflection [12]. Within this paradigm, *attitude* and *motivation* are core components of successful learning [13].

Attitude—comprising cognitive, affective, and behavioral dimensions—plays a significant role in academic achievement and professional development [11, 14]. Motivation, the internal drive that stimulates action, has been identified as a critical predictor of learning persistence and performance [15]. According to Deci and Ryan’s Self-Determination Theory , motivation varies in autonomy and includes amotivation, extrinsic motivation, and intrinsic motivation [16, 17]. A growing body of research confirms that higher motivation and positive attitudes enhance academic engagement, satisfaction, and career success [11, 18–20]. However, in Iran, rehabilitation is not typically students’ first-choice field. Many students enter based on national exam rankings rather than personal interest, often leading to limited pre-entry knowledge and lack of professional identity.

Promoting knowledge, positive attitudes, and intrinsic motivation throughout training is therefore essential for improving educational quality and workforce commitment. This study aimed to examine the effects of the Principles of Rehabilitation course on undergraduate students’ perceived knowledge, attitude, and motivation toward their chosen profession. Additionally, it assessed how early clinical exposure among third-year students influences these factors. Using the validated Rehabilitation Sciences perceived Knowledge, Attitude, and Motivation (RS-KAM) questionnaire, this study seeks to inform curricular strategies that enhance student engagement, professional commitment, and satisfaction among Iranian rehabilitation students.

## Methods

### Participants

A total of 184 undergraduate students from the School of Rehabilitation Sciences at Tabriz University of Medical Sciences were recruited. The sample comprised 107 first-year students and 77 third-year students enrolled in physiotherapy, occupational therapy, speech therapy, and audiology programs. Participation was voluntary, and written informed consent was obtained from all participants prior to data collection.

### Study design

This study employed a quasi-experimental pretest–posttest design to evaluate the effect of the Principles of Rehabilitation course on first-year students’ perceived knowledge, attitudes, and motivation. Randomization was not feasible because course scheduling within each discipline was determined by the respective academic departments, and all courses were delivered in accordance with a nationally approved curriculum mandated by the Ministry of Health, which did not allow for modification or reassignment of course delivery. Therefore, physiotherapy students, who were scheduled to receive the course in a subsequent semester, were selected as control group. These students were assessed over the same time period but did not receive the intervention during the study. First-year students completed the RS-KAM questionnaire at two time points: at the beginning (pretest) and at the end (posttest) of the semester. In addition, third-year students completed the questionnaire once to enable cross-sectional comparisons related to academic progression and early clinical exposure.

### Educational intervention

The Principles of Rehabilitation course is a compulsory component of the undergraduate curriculum delivered during the first academic year. The intervention consisted of eight structured sessions, each lasting approximately 60 to 120 min, delivered over one academic semester. A standardized instructional framework was applied to ensure consistency across disciplines and instructors. The course adopted a blended learning approach integrating didactic lectures for core theoretical content, team-based learning to promote active engagement, and supervised field visits to rehabilitation centers to provide experiential learning opportunities. These field visits were designed to familiarize students with real-world rehabilitation settings, interdisciplinary collaboration, and the use of assistive technologies in clinical practice.

### Data collection instrument

Data were collected using the RS-KAM questionnaire, a newly developed and preliminarily validated instrument designed to assess undergraduate students’ perceived knowledge, attitudes, and motivation toward rehabilitation sciences (Maleki M, Amirshakeri B, Jahan A, Soltani-zangbar H: Psychometric Properties of Perceived-Knowledge, Attitude and Motivation Questionnaire toward Rehabilitation Sciences (RS-KAM Questionnaire), submitted). Content validity was established through expert panel evaluation, yielding a Content Validity Ratio of 0.82 and Content Validity Index values ranging from 0.97 to 0.99. Construct validity was supported by exploratory factor analysis, with a Kaiser–Meyer–Olkin value of 0.80 and a significant Bartlett’s test of sphericity (*p* < 0.001), identifying a three-factor structure consistent with the theoretical framework and explaining 53.47% of the total variance. The instrument demonstrated acceptable reliability, with an overall Cronbach’s alpha of 0.847 and subscale coefficients ranging from 0.75 to 0.88. Test–retest reliability over a 1–2-week interval indicated adequate stability, with intraclass correlation coefficients ranging from 0.75 to 0.79. The questionnaire consists of two sections. The first section collects demographic and background information, including age, gender, academic major, socioeconomic status, and prior familiarity with rehabilitation sciences. The second section includes 29 items assessing perceived knowledge, attitudes, and motivation. Items 1 to 10 were used for descriptive purposes only and were excluded from inferential analyses. The remaining items were scored using a Visual Analog Scale ranging from 0 to 10, with higher scores indicating greater perceived knowledge, more positive attitudes, or higher motivation. Composite scores were calculated for each domain based on the validated factor structure. The perceived knowledge domain included seven items with a possible score range of 0 to 70, the attitude domain included eight items with a range of 0 to 80, and the motivation domain included four items with a range of 0 to 40. Higher composite scores indicate higher levels of the respective constructs. Given the preliminary validation status of the instrument, findings should be interpreted as reflecting perceived rather than objective measures. The questionnaire is provided in Appendix 1 (Persian version) and Appendix 2 (English translation).

### Data analysis

Descriptive statistics were used to summarize participants’ demographic characteristics and baseline scores. The primary analysis focused on between-group comparisons of change scores, calculated as the difference between posttest and pretest values, between intervention and control groups across the three main outcomes of perceived knowledge, attitude, and motivation. To further examine the effect of the intervention across study programs, mixed-model analysis of variance was conducted with time as the within-subject factor and group as the between-subject factor. Secondary analyses included paired t-tests for within-group comparisons, independent t-tests for comparisons between first-year and third-year students, and Spearman’s correlation coefficients to assess relationships among perceived knowledge, attitude, and motivation. These secondary analyses were considered exploratory in nature. Given the number of statistical tests performed, the potential for Type I error inflation should be considered when interpreting these findings. All statistical analyses were performed using SPSS version 25, and statistical significance was set at *p* < 0.05.

### Ethical considerations

The study was approved by the Ethics Committee of Tabriz University of Medical Sciences (Approval No. IR.TBZMED.REC.1403.635) and conducted in accordance with the principles of the Declaration of Helsinki. Participation was voluntary, and all data were anonymized to ensure confidentiality.

## Results

### Descriptive analysis of RS-KAM questionnaire responses

Table 1 presents the demographic characteristics of the 184 participating students. The mean age was 21.5 (± 3.6) years, and 57.6% of participants were female. A total of 19% reported previous academic education unrelated to rehabilitation sciences, and 58% reported prior experience with rehabilitation services. Approximately 34.2% had received mentorship or counseling regarding their field of study prior to university entry. Socioeconomic status was reported as poor by 6% of participants, moderate by 52.7%, and good by 41.3%. Baseline RS-KAM scores and descriptive comparisons are summarized in Table 2. First-year students showed increases in perceived knowledge scores over time. Changes in attitudes were modest, while motivation scores exhibited slight declines. Third-year students generally demonstrated higher perceived knowledge and attitude scores compared with first-year students, whereas motivation scores tended to be lower.

**Table 1:** Participant characteristics and descriptive statistics of RS-KAM questionnaire (*N* = 184)

Variable	Category	Value
Age (years)	Mean ± SD	21.5 ± 3.6
Gender	Male	41.8%
	Female	57.6%
Major	Audiology	16.8%
	Physical Therapy	37.5%
	Occupational Therapy	27.2%
	Speech Therapy	18.5%
Socio-economic status (SES)	Poor	6.0%
	Moderate	52.7%
	Good	41.3%
Mentor counseling	Yes	34.2%
	No	63.8%
National exam entrance frequency	Mean ± SD	2.2 ± 1.0
Rehabilitation service history	Yes	58.0%
	No	41.8%
Other academic education	Yes	19.0%
	No	81.0%

*SD* Standard deviation, *SES* Socio-economic status, Mentor counseling refers to receiving guidance or information about rehabilitation-related fields; National exam entrance frequency indicates the number of times an individual is allowed to take the national examination; Other academic education denotes prior academic experience in fields other than the current discipline

**Table 2:** Mean ± SD of RS-KAM subscale scores by field of study and academic year

Subscale	Group	Audiology	Physical Therapy	Occupational Therapy	Speech Therapy
Knowledge	First year – Pre	36 ± 10	31 ± 12	27 ± 12	35 ± 11
	First year – Post	47 ± 8	38 ± 12	42 ± 8	44 ± 12
	Δ score	+ 11	+ 7	+ 15	+ 9
	Third year	40 ± 7	41 ± 11	42 ± 10	46 ± 13
Attitude	First year – Pre	61 ± 12	64 ± 13	61 ± 11	58 ± 8
	First year – Post	66 ± 10	58 ± 12	62 ± 7	57 ± 9
	Δ score	+ 5	− 6	+ 1	− 1
	Third year	61 ± 8	64 ± 11	66 ± 11	64 ± 9
Motivation	First year – Pre	27 ± 7	28 ± 5	26 ± 5	27 ± 5
	First year – Post	25 ± 6	25 ± 6	22 ± 6	24 ± 6
	Δ score	− 2	− 3	− 4	− 3
	Third year	18 ± 8	21 ± 5	21 ± 8	18 ± 6

*RS-KAM* Rehabilitation Sciences Knowledge, Attitude, and Motivation questionnaire, *A* Audiology, *P* Physical Therapy, *O* Occupational Therapy, *S* Speech Therapy, *K* Perceived knowledge, *A* Attitude toward rehabilitation sciences and academic education; M = Motivation and expectations, Δ score represents the change from pre-intervention to post-intervention (Post − Pre); Positive values indicate improvement, while negative values indicate a decrease; First-year students were assessed before and after completion of the Principles of Rehabilitation course. Third-year students were assessed once for comparison

### Case–control analysis of the impact of the course (Primary analysis)

The primary analysis examined between-group differences in change scores (posttest minus pretest) between the intervention group (audiology, speech therapy, and occupational therapy; *n* = 60) and the control group (physiotherapy; *n* = 22). A statistically significant difference was observed for attitude change, with the intervention group demonstrating a positive mean change (Δ = 1.5) compared with a decrease in the control group (Δ = − 7.6) [t(80) = 3.3, *p* = 0.001].

**Fig. 1:**
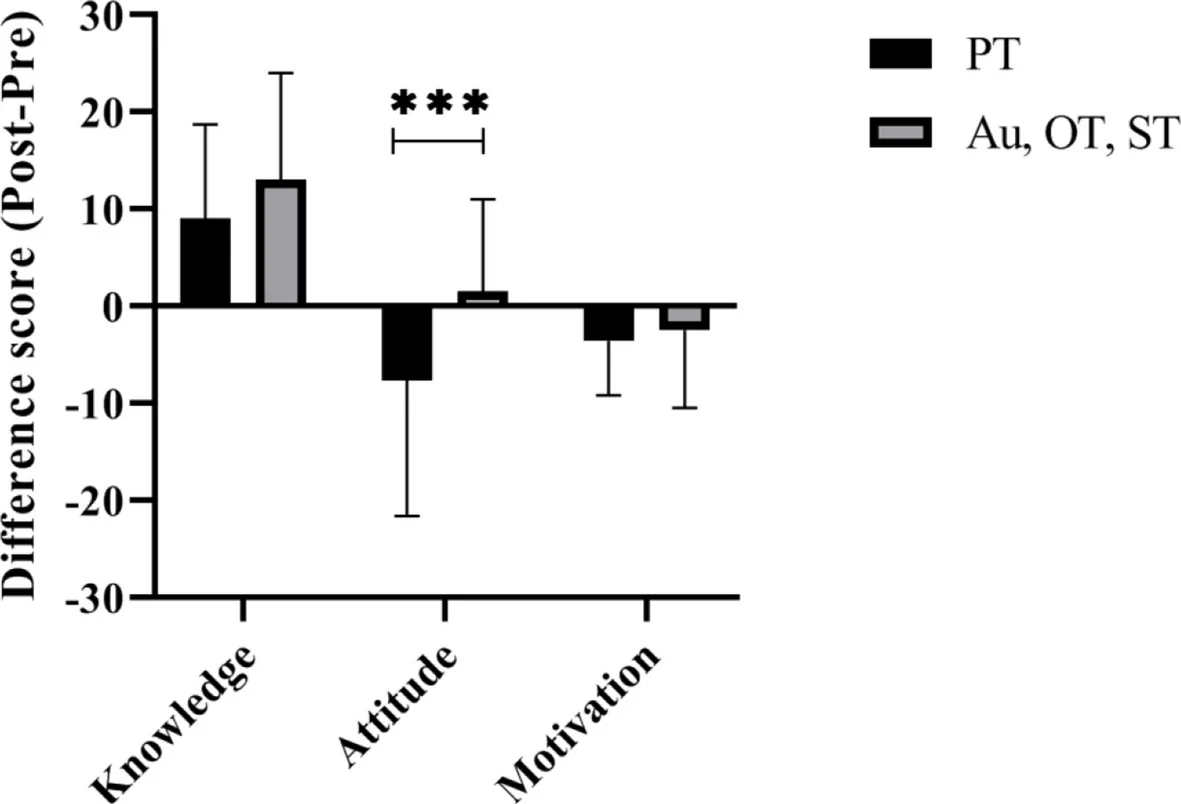
Comparison of difference scores in perceived knowledge, attitudes, and motivation (KAM) toward rehabilitation sciences between intervention (Audiology, Occupational Therapy, Speech Therapy) and control (Physical Therapy) groups. *** *p* < 0.001 indicates statistically significant differences

For perceived knowledge, both groups showed increases over time; however, the between-group difference in change scores was not statistically significant (intervention: Δ = 13; control: Δ = 9; t(80) = 1.55, *p* = 0.12). Similarly, motivation decreased in both groups, and the between-group comparison did not reach statistical significance (intervention: Δ = − 2.5; control: Δ = − 3.6; t(79) = 0.55, *p* = 0.58). These findings indicate that, among the three outcome domains, only attitude demonstrated a statistically significant difference between intervention and control groups (Fig. 1).

### Within-group changes in first-year students (Exploratory analysis)

**Fig. 2:**
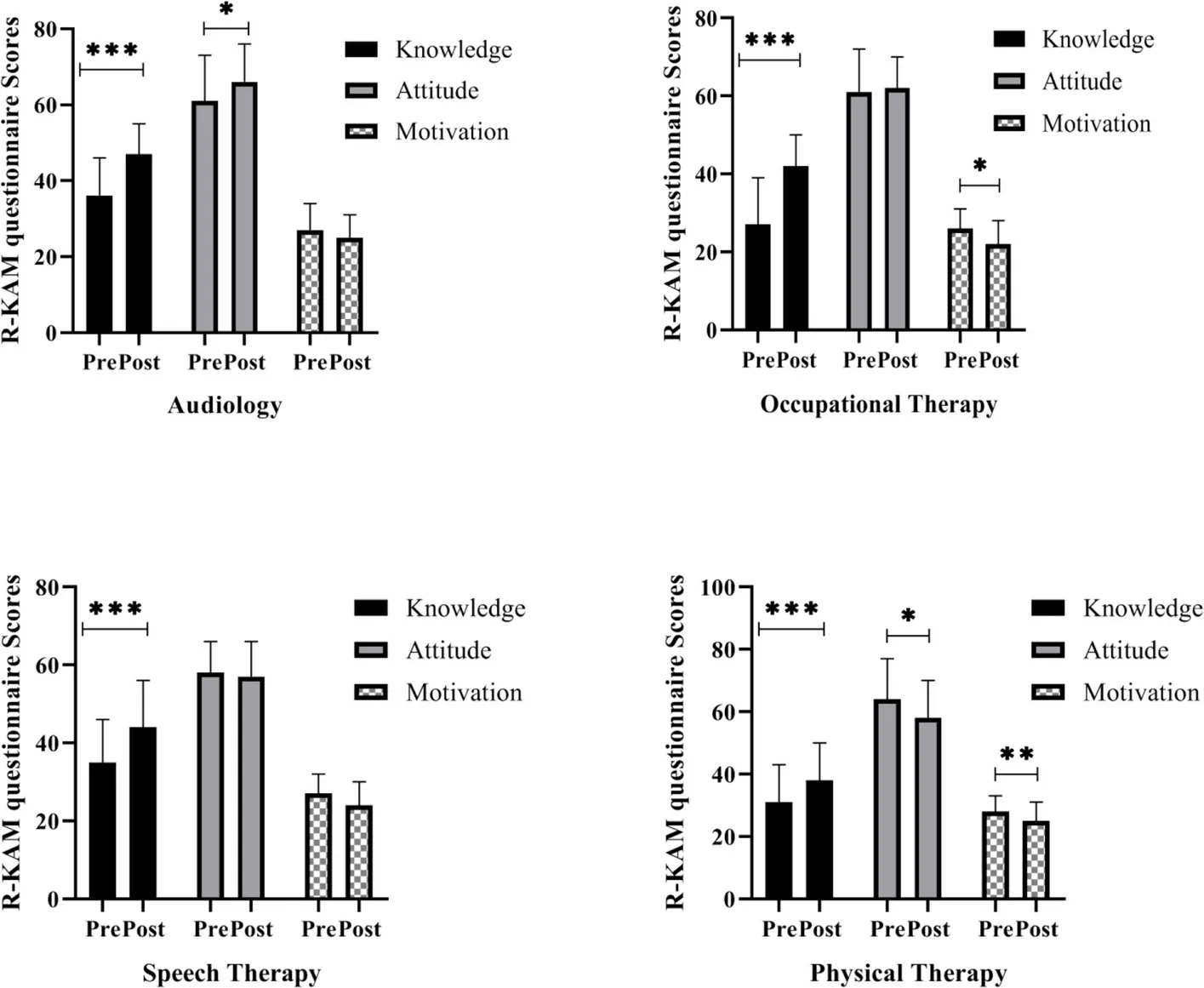
Comparison of scores in perceived knowledge, attitudes, and motivation (KAM) among first-year students, before and after completion of the ‘Principles of Rehabilitation’ course. Significance levels: *p < 0.05; **p < 0.01; **p < *0.001*

Within-group changes over time were examined descriptively among first-year students in the intervention programs. A mixed-model ANOVA indicated a significant main effect of time [F(1, 57) = 18.0, *p* < 0.001, η2 = 0.24], along with significant interaction effects for Time × Subtests and Time × Major × Subtests, while the Time × Major interaction was not significant. Assumptions for ANOVA, including equality of covariance matrices and sphericity, were met. Paired t-test analyses showed that perceived knowledge increased significantly within the intervention group (Δ = 13.3, *p* < 0.001), while motivation decreased modestly (Δ = − 2.5, *p* = 0.02). The change in attitude was not statistically significant (Δ = 1.5, *p* = 0.22). The total RS-KAM score showed a significant increase over time (Δ = 12.3, *p* < 0.01). Subgroup analyses by academic major suggested variability in patterns of change across disciplines. However, these analyses were exploratory and are presented for descriptive purposes (Fig. 2). In the control group, perceived knowledge also increased significantly (Δ = 9.0, *p* < 0.001), while decreases were observed in both attitude and motivation.

### Cross-sectional comparison between first-year and third-year students

**Fig. 3:**
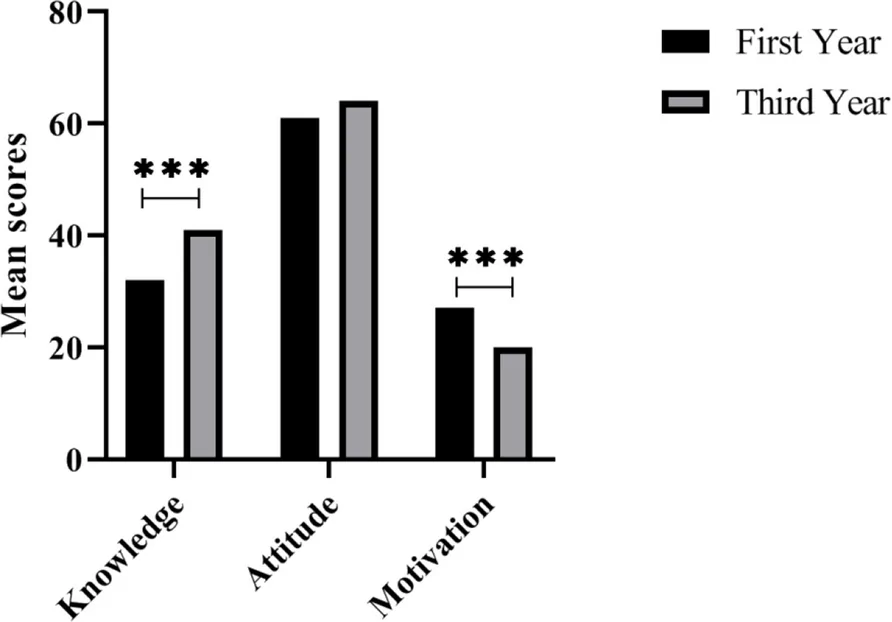
Comparison of perceived knowledge, attitudes, and motivation (KAM) scores between first-year and third-year students across rehabilitation majors. The figure displays cross-sectional differences in perceived knowledge, attitude, and motivation among students at different stages of training. ***p < 0.001, indicating statistically significant differences

Independent t-tests were conducted to compare RS-KAM scores between first-year and third-year students. Third-year students demonstrated significantly higher perceived knowledge than first-year students [mean difference = 9.4, t(155) = 5.09, *p* < 0.001]. In contrast, motivation scores were significantly lower among third-year students [mean difference = − 6.3, t(108) = 6.12, *p* < 0.001]. No statistically significant difference was observed in attitude scores between the two groups [t(155) = 1.6, *p* = 0.11] (Fig. 3).

### Effect of pre-university informational counseling (Exploratory analysis)

**Fig. 4:**
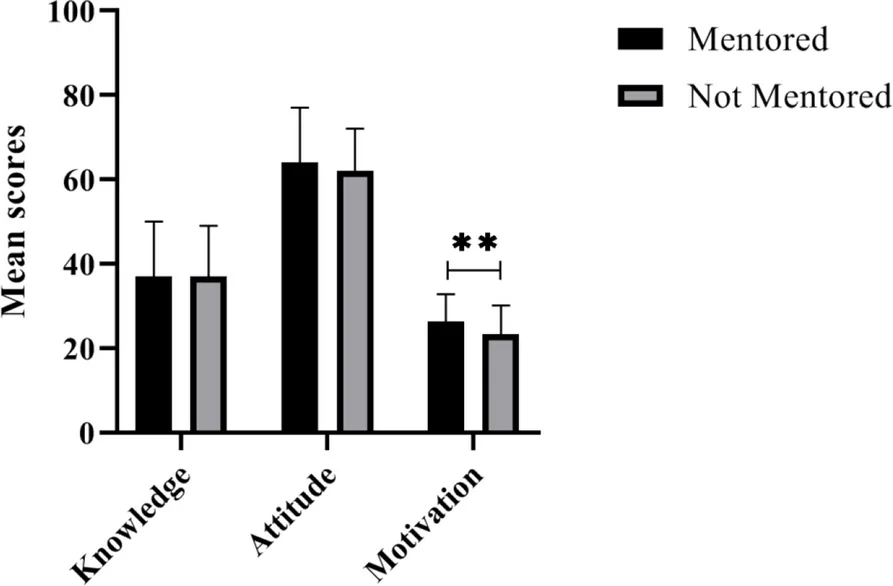
Mean perceived knowledge, attitudes, and motivation (KAM) scores for mentored and non‑mentored student groups. The figure compares perceived knowledge, attitude, and motivation between students who participated in mentoring activities and those without mentoring exposure. **p < 0.01, indicating statistically significant differences

An independent t-test was conducted to examine the association between receiving informational counseling prior to university entry and RS-KAM outcomes among first-year students. Students who had received counseling demonstrated significantly higher motivation scores compared to those who had not [t(151) = 2.64, *p* = 0.009] (Fig. 4). No significant differences were observed for perceived knowledge or attitude.

### Interrelationships among perceived knowledge, attitude, and motivation

Spearman correlation analysis showed significant positive associations among the three constructs in first-year students. Perceived knowledge was weakly to moderately correlated with attitude (rho = 0.25, *p* = 0.01) and motivation (rho = 0.31, *p* = 0.002), and attitude was also positively correlated with motivation (rho = 0.35, *p* < 0.001). Among third-year students, a significant positive correlation was observed only between perceived knowledge and attitude (rho = 0.37, *p* = 0.003), while the correlations between perceived knowledge and motivation (rho = 0.18, *p* = 0.15) and between attitude and motivation (rho = 0.13, *p* = 0.30) were not statistically significant (Table 3). These findings suggest differences in the pattern of associations across academic stages; however, these analyses are exploratory.

**Table 3:** Spearman’s rho (ρ) correlations and p-values among RS-KAM subscales

Variables	First-year ρ (p)	Third-year ρ (p)
Knowledge – Attitude	**0.25 (0.01)**	**0.37 (0.003)**
Knowledge – Motivation	**0.31 (0.002)**	0.18 (0.15)
Attitude – Motivation	**0.35 (< 0.001)**	0.13 (0.30)

Spearman’s rho was used to assess correlations between RS-KAM subscales; K = Perceived knowledge; A = Attitude toward rehabilitation professions; M = Motivation to pursue rehabilitation-related education or careers; Statistically significant correlations (*p* < 0.05) are presented in bold

## Discussion

This study examined the effects of a foundational educational course and early clinical exposure on undergraduate rehabilitation students’ perceived knowledge, attitude, and motivation. The primary finding, based on between-group comparisons, was that only attitude demonstrated a statistically significant difference between the intervention and control groups, while no significant between-group differences were observed for perceived knowledge or motivation.

The observed improvement in attitude among students who completed the Principles of Rehabilitation course highlights the potential role of structured educational experiences in shaping early professional perspectives. In contrast, the decline in attitude observed in the control group may reflect the absence of such structured exposure during the same period. These findings are consistent with existing literature emphasizing the importance of early professional socialization in the development of professional identity [21]. However, given the quasi-experimental design and the reliance on self-reported outcomes, these results should be interpreted cautiously and do not necessarily reflect objective changes in professional competence or behavior.

In contrast to attitude, perceived knowledge increased over time in both intervention and control groups, with no statistically significant difference between groups. These findings should be interpreted as changes in perceived knowledge rather than objective knowledge, as self-reported measures are inherently subjective and may not accurately reflect actual performance or competence [22]. Furthermore, improvements in perceived knowledge may also be influenced by informal and experiential learning processes occurring outside formal instruction [23].

Motivation decreased modestly in both groups, and no between-group difference was observed. This pattern suggests that the decline in motivation may not be directly attributable to the intervention. Instead, it may reflect broader transitional challenges experienced by students during the early stages of university education [24]. At the beginning of a university program, students face several challenges, including adapting to new learning environments, adjusting to learning strategies that may not be well suited to rigorous scientific study, managing broader life changes, and coping with potential disappointment when high expectations are not met, all of which can affect their motivational states [25–29]. This interpretation can be supported by Person–Environment Fit Theory, which suggests that a mismatch between individuals’ expectations and environmental demands may negatively affect motivation and adjustment [30].

Within-group analyses showed that first-year students in the intervention group experienced increases in perceived knowledge and a small decline in motivation over time. However, as no corresponding between-group differences were identified, these changes should be interpreted as descriptive patterns rather than evidence of intervention effects.

Cross-sectional comparisons between first-year and third-year students revealed that students with early clinical exposure reported higher perceived knowledge but lower motivation. The transition from preclinical to clinical training has been described in previous studies as a potentially challenging phase, often accompanied by increased workload, emotional demands, and uncertainty about professional roles [31, 32]. From a theoretical perspective, Self-Determination Theory provides a useful framework for interpreting these findings, suggesting that motivation is influenced by perceived autonomy, competence, and relatedness. Early exposure to the complexities of clinical practice may temporarily reduce intrinsic motivation as students adjust their expectations and confront real-world challenges. However, given the self-reported nature of the outcomes and the cross-sectional design, these interpretations should be considered exploratory rather than causal. Prior studies have similarly reported that the transition to clinical training can be experienced as stressful and may lead to temporary reductions in motivation, although motivation may recover over time as students develop greater competence and self-efficacy [18, 33].

The finding that students who received pre-university informational counseling demonstrated higher motivation suggests that early exposure to accurate information about academic programs may help shape expectations and support engagement. This finding aligns with prior research emphasizing the role of career awareness in supporting and enhancing students’ motivation [34].

Finally, the observed correlations among perceived knowledge, attitude, and motivation, particularly in first-year students, suggest potential interrelationships among these constructs during early stages of training [34]. The weaker associations observed in third-year students may reflect increasing complexity as students integrate theoretical knowledge with clinical experience. These findings should be considered exploratory and interpreted as hypothesis-generating rather than definitive evidence, warranting further investigation through longitudinal and controlled study designs.

### Limitations and implications

This study has several limitations. The quasi-experimental design without randomization limits causal inference, and the possibility of selection bias cannot be fully excluded. In addition, the relatively small subgroup sizes in some analyses require cautious interpretation of subgroup findings. The use of self-reported measures may not accurately reflect objective knowledge, clinical competence, or behavioral outcomes. Therefore, the findings should be interpreted as reflecting students’ perceived educational experiences rather than objective educational effectiveness. Furthermore, the RS-KAM questionnaire should be considered a context-specific and still-developing educational measure. Although the instrument demonstrated acceptable preliminary psychometric properties in the present sample, interpretation of composite scores and broader generalizability should be approached cautiously. Additional validation studies using confirmatory factor analysis, independent samples, and diverse educational settings are recommended.

The study was conducted at a single institution, which may further limit the generalizability of the findings. Future research should employ longitudinal, multi-center, and controlled study designs and incorporate mixed-method approaches to better understand changes in motivation, professional development, and educational outcomes over time.

From an educational perspective, the findings suggest that structured introductory courses may contribute to shaping students’ professional attitudes and perceived preparedness. Additionally, incorporating supportive strategies such as mentoring, early clinical exposure, and active learning approaches may help address potential declines in motivation during early academic transitions.

## Conclusion

This study found that participation in the Principles of Rehabilitation course was associated with a significant improvement in students’ attitudes compared with the control group, while no between-group differences were observed for perceived knowledge or motivation. Improvements in perceived knowledge occurred in both groups, suggesting that these changes cannot be attributed solely to the structured course and should not be interpreted as evidence of objective educational effectiveness. A decline in motivation was observed across both early academic and clinical stages, indicating that motivational changes may reflect broader transitional challenges rather than the effect of a single educational intervention. Supporting student motivation alongside cognitive development may therefore be an important consideration in rehabilitation education.

## Supplementary Information

Supplementary Material 1.

Supplementary Material 2.

## Data Availability

The datasets generated and/or analyzed during the current study are available from the corresponding author on reasonable request.
