# Supplementary material for: Evaluating the effects of the educational course on undergraduate students’ perceived knowledge, attitude, and motivation: a quasi-experimental study in Iran
Source: BMC Med Educ. 2026 Jul 22;26:1668. doi: 10.1186/s12909-026-09915-z (PMC13678942; doi:10.1186/s12909-026-09915-z)
Supplement: Supplementary file 1 [file 12909_2026_9915_MOESM1_ESM.docx]

**توجه: در پرسشنامه حاضر 29 سوال با هدف بررسی سطح دانش ، نگرش و انگیزه دانشجویان جدیدالورود به رشته تحصیلی خود مطرح شده است. لطفا با دقت پاسخ دهید. اطلاعات شما به طور محرمانه نگه داری میشود و صرفا در راستای پژوهش و ارتقای کیفیت آموزش استفاده خواهد شد.**

**اطلاعات دموگرافیک و عمومی**

نام و نام خانوادگی: سن: جنسیت: رشته تحصیلی:

وضعیت اجتماعی-اقتصادی خانواده:

ضعیف (میانگین درآمد ماهانه خانواده کمتر از 12 میلیون تومان)

متوسط (میانگین درآمد ماهانه خانواده بین 12 تا 20 میلیون تومان)

خوب	(میانگین درآمد ماهانه خانواده بیشتر از 20 میلیون تومان)

- آیا در خصوص رشته های توانبخشی منتور یا مشاور مدرسه اطلاعاتی در اختیارتان قرار دادند؟	بلی	خیر
- از چه طریق با رشته کنونی خود آشنا شدید؟

مشاور کنکور	دوستان و آشنایان	تحقیق توسط خودتان	تطابق با رتبه کنکور	سایر موارد.........

- آیا اولین سالی است که در کنکور شرکت کردید؟	اگر خیر چندمین بار است؟
- آیا تجربه استفاده از خدمات توانبخشی توسط خودتان یا اقوام نزدیک را دارید؟	بلی	خیر	چه نوع خدماتی؟
- آیا تجربه تحصیلات اکادمیک در رشته های دیگر یا کشور های دیگر دارید؟ اگر بلی چه رشته ای و کدام کشور؟

**پرسشنامه محقق ساخته جهت بررسی دانش ادراک شده، انگیزه و نگرش**

**الف) بخش سوالات کیفی**

1. اهمیت رشته های توانبخشی را به اندازه سایر رشته های علوم پزشکی می دانم. بلی	خیر
2. پرستیژ (موقعیت و جایگاه) اجتماعی رشته های توانبخشی در جامعه مورد قبول من است. بلی	خیر
3. به ادامه تحصیل در رشته خود علاقمندم. بله	خیر
4. از بازار کار رشته های توانبخشی مطلع هستم. بله	خیر
5. برای ایجاد پیشرفت در خدمات توانبخشی انگیزه کافی دارم. بله	خیر
6. از نظر روحی و روانی خود را مناسب فعالیت در رشته های توانبخشی می دانم. بله	خیر
7. از نظر جسمی خود را مناسب فعالیت در رشته های توانبخشی می دانم. بله	خیر
8. با انجمن ها و نهادهای مرتبط با خدمات توانبخشی آشنایی دارم. بلی	خیر
9. آیا مانعی در مسیر پیشرفت تحصیلی خود احساس میکنید؟	بلی	خیر	اگر بله چه مانعی؟
10. بعد از اتمام دوره کارشناسی تمایل دارید در همین رشته ادامه تحصیل دهید یا وارد بازار کار شوید یا هردو؟

**ب) بخش سوالات کمی**

**لطفا به سوالات زیر از صفر تا 10 نمره بدهید (صفر به معنی حداقل مقدار ممکن و 10 نشانگر حداکثر مقدار ممکن است)**

11. دانش و اطلاعات شما از علوم (رشته های) توانبخشی شامل شنوایی شناسی، گفتاردرمانی ، کاردرمانی ، فیزیوتراپی ، بینایی سنجی و اعضای مصنوعی و مدیریت توانبخشی چه میزان است؟

12. دانش و اطلاعات شما از **رشته تخصصی خودتان** چقدر است؟

13. به نظر شما **نقش متخصصان رشته های توانبخشی** در سلامت جامعه چقدر است؟

14. به نظر شما نقش توانبخشی در افزایش کیفیت زندگی **افراد جامعه** چقدر است؟

15. به نظر شما نقش توانبخشی در افزایش کیفیت زندگی **مراجعین** چقدر است؟

16. **میزان انگیزه** خود را جهت تحصیل در **رشته های توانبخشی** چگونه ارزیابی میکنید؟

17. میزان **رضایت** شما از رشته ای که قبول شده اید چقدر است؟

18. به نظر شما رشته ای که قبول شده اید **انتظارات شما را از تحصیل دانشگاهی** برآورده خواهد کرد؟

19. به نظر شما رشته ای که قبول شده اید **انتظارات شما را از شغل آینده** برآورده خواهد کرد؟

20. تا چه حد انتظارات شما از دانشگاه (شامل **فضای فیزیکی**) محقق شده است؟

21. تا چه حد انتظارات شما از دانشگاه (شامل **امکانات** **آموزشی و رفاهی**) محقق شده است؟

22. تا چه حد انتظارات شما از دانشگاه (**شامل نیروی انسانی و اساتید**) محقق شده است؟

23. میزان آشنایی شما با حمایت های قانونی از رشته های توانبخشی چقدر است؟

24. همکاری بین رشته های توانبخشی را برای بهبود بیماران چقدر مهم می دانید؟

25. در مورد تاثیر کار تیمی در توانبخشی بیماران چقدر اطلاعات دارید؟

26. از **تاریخچه توانبخشی** در ایران چقدر اطلاع دارید؟

27. از **تاریخچه توانبخشی در جهان** چقدر اطلاع دارید؟

28. میزان **آگاهی شما از رابطه پیشگیری، درمان و توانبخشی** چقدر است؟

29. به چه میزان مقطع کارشناسی را برای ارائه خدمات مطلوب توانبخشی کافی می دانید؟
