# Supplementary material for: Evaluating the effects of the educational course on undergraduate students’ perceived knowledge, attitude, and motivation: a quasi-experimental study in Iran
Source: BMC Med Educ. 2026 Jul 22;26:1668. doi: 10.1186/s12909-026-09915-z (PMC13678942; doi:10.1186/s12909-026-09915-z)
Supplement: Supplementary file 2 [file 12909_2026_9915_MOESM2_ESM.docx]

**Perceived-Knowledge, Attitude and Motivation Questionnaire toward Rehabilitation Sciences (RS-KAM questionnaire)**

**Note:** This questionnaire includes 29 questions aimed at assessing the level of perceived-knowledge, attitude, and motivation of rehabilitation sciences students regarding their chosen field of study. Please answer carefully. Your information will be kept confidential and will only be used for research and improving the quality of education.

**Demographic and General Information**

- **Name and surname:** .............................................
- **Age:** ..............
- **Gender:** ☐ Male ☐ Female ☐ Other
- **Field of study:** .............................................

**Family socio-economic status:**
☐ Weak (average monthly family income less than 120 million Rials)
☐ Moderate (average monthly family income between 120–200 million Rials)
☐ Good (average monthly family income more than 200 million Rials)

- Did your school mentor/counselor provide information about rehabilitation fields?
  ☐ Yes ☐ No
- How did you become familiar with your current field of study?
  ☐ University entrance exam counselor
  ☐ Friends and acquaintances
  ☐ Self-research
  ☐ Based on entrance exam ranking
  ☐ Other: ............................
- Is this your first time taking the university entrance exam?
  ☐ Yes ☐ No → If no, how many times? .............
- Do you or your close relatives have experience using rehabilitation services?
  ☐ Yes ☐ No	If yes, please specify: ............................
- Do you have academic experience in other fields or other countries? ☐ Yes ☐ No
  If yes, please specify: Field ............. Country .............

**Perceived-Knowledge, Attitude, and Motivation**

**Part A – Yes/No Questions (Quantitative Section)**

1. I consider the importance of rehabilitation fields equal to other medical sciences.
   ☐ Yes ☐ No
2. The social prestige (status and position) of rehabilitation fields is acceptable to me.
   ☐ Yes ☐ No
3. I am interested in continuing my studies in my field.
   ☐ Yes ☐ No
4. I am aware of the job market of rehabilitation fields.
   ☐ Yes ☐ No
5. I am motivated enough to contribute to the improvement of rehabilitation services.
   ☐ Yes ☐ No
6. I consider myself mentally and psychologically suitable for working in rehabilitation fields. ☐ Yes ☐ No
7. I consider myself physically suitable for working in rehabilitation fields.
   ☐ Yes ☐ No
8. I am familiar with associations and organizations related to rehabilitation services.
   ☐ Yes ☐ No
9. Do you feel any obstacles in your academic progress?
   ☐ Yes ☐ No	If yes, what kind of obstacle? ............................
10. After completing your bachelor’s degree, you prefer to:
    ☐ Continue studying in the same field
    ☐ Enter the job market
    ☐ Both

**Part B – Visual Analogue Scale (VAS) Questions (Quantitative Section)**

*(Please mark a point on the line that best represents your answer. The left side means the minimum value, and the right side means the maximum value.)*

**11.** Your knowledge of rehabilitation sciences (audiology, speech therapy, occupational therapy, physiotherapy, optometry, prosthetics, rehabilitation management).
0 ──────────────────────────────── 10

**12.** Your knowledge of your own field of specialization.
0 ──────────────────────────────── 10

**13.** The role of rehabilitation specialists in public health.
0 ──────────────────────────────── 10

**14.** The role of rehabilitation in improving quality of life in society.
0 ──────────────────────────────── 10

**15.** The role of rehabilitation in improving quality of life of patients/clients.
0 ──────────────────────────────── 10

**16.** Your motivation for studying in rehabilitation fields.
0 ──────────────────────────────── 10

**17.** Your satisfaction with the field you were admitted to. 0 ──────────────────────────────── 10

**18.** Do you think your field of study will meet your expectations for academic education?
0 ──────────────────────────────── 10

**19.** Do you think your field of study will meet your expectations for your future career?
0 ──────────────────────────────── 10

**20.** To what extent have your expectations from the university (physical environment) been met?
0 ──────────────────────────────── 10

**21.** To what extent have your expectations from the university (educational and welfare facilities) been met?
0 ──────────────────────────────── 10

**22.** To what extent have your expectations from the university (faculty and staff) been met?
0 ──────────────────────────────── 10

**23.** Your familiarity with legal supports for rehabilitation fields.
0 ──────────────────────────────── 10

**24.** The importance of inter-professional collaboration among rehabilitation fields for patient improvement.
0 ──────────────────────────────── 10

**25.** Your knowledge about the impact of teamwork on patient rehabilitation.
0 ──────────────────────────────── 10

**26.** Your knowledge about the history of rehabilitation in Iran.
0 ──────────────────────────────── 10

**27.** Your knowledge about the history of rehabilitation in the world.
0 ──────────────────────────────── 10

**28.** Your awareness of the relationship between prevention, treatment, and rehabilitation.
0 ──────────────────────────────── 10

**29.** To what extent do you consider a bachelor’s degree sufficient for providing proper rehabilitation services?
0 ──────────────────────────────── 10
